# *Bacillus silvicola* sp. nov., a new species within the *Bacillus cereus* group isolated from hardwood forest soil in Maryland, USA

**DOI:** 10.1038/s41598-026-52504-9

**Published:** 2026-05-13

**Authors:** Holly P. Bartholomew, Michael E. Sparks, Daniel Kuhar, Ashaki Mitchell, Robert R. Farrar, Dawn E. Gundersen-Rindal, Michael B. Blackburn

**Affiliations:** Invasive Insect Biocontrol and Behavior Laboratory, USDA-ARS, Henry A. Wallace Beltsville Agricultural Research Center, Beltsville, MD 20705 USA

**Keywords:** *Bacillus silvicola*, Whole-genome sequencing, Species nova, *Bacillus cereus senso lato*, Parasporal crystal, Biotechnology, Microbiology, Molecular biology

## Abstract

A single bacterial strain, IBL03679^T^, from forest soil in Maryland, USA in 2009 was originally identified as belonging to the *Bacillus cereus senso lato* group through multi-locus sequence analysis. Here, further identification efforts with genomic and phenotypic methods demonstrate that this strain belongs to a new species. Phylogenetic analysis using whole-genome sequencing suggested the most closely related species is *Bacillus proteolyticus*. ANIb and dDDH values confirmed species differentiation with values below conventional 95–96% and 70% thresholds, respectively. Genomic comparisons with five other *Bacillus* strains with the same sequence type also group into the proposed species nov. It is a gram-stain positive, facultative anaerobe, endospore and parasporal crystal forming rod-shaped bacterium. Physiological characteristics, such as casein hydrolysis and lecithinase production, and major fatty acids identified (C_16:0_, iso-C_15:0_, and iso-C_17:0_), support affiliation with other *Bacillus* species. The polar lipid profile was primarily diphosphatidylglycerol (DPG), phosphatidylethanolamine (PE), and phosphatidylglyercol (PG); the peptidoglycan type was A1γ meso-Dpm – direct (A31); and the sole respiratory quinone was menaquinone-7. Biochemical testing revealed distinguishing test results for strain IBL03679^T^ through inability to metabolize D-cellobiose and D-saccharose, and utilization of potassium gluconate. Collectively, the genotypic and phenotypic data demonstrate that IBL03679^T^ belongs to a novel species. We propose the name *Bacillus silvicola* sp. nov. for this taxon with the type strain IBL03679^T^ (= NRRL B-65743^T^, = CCUG 78293^T^).

**Supplementary Information:**

The online version contains supplementary material available at 10.1038/s41598-026-52504-9.

## Introduction

 The genus *Bacillus* contains over 100 species, with many diverse characteristics^1^. Within the genus are a sub-classification for the *Bacillus cereus* group, designated *Bacillus cereus senso lato*. Thus far, the group is comprised of 20 closely related, but distinct, species^2–4^. Species within the group are ubiquitous in diverse environmental niches, including soil, water, animal, plant, and even food sources^2,4,5^. Some species within the group include *Bacillu*s *anthracis* as a human pathogen, *Bacillus cereus* associated with foodborne illness, *Bacillus nitratireductans*, *Bacillus proteolyticus*, and *Bacillus thuringiensis* (Bt), which is well known for having insecticidal activity^3–7^.

The strain described here, IBL03679^T^ (syn. IBL 3679), was originally isolated in 2009 from a soil sample obtained from a mature hardwood forest in Maryland, USA^7^. It was one of 121 strains chosen for study because it could form parasporal crystals, which could indicate a potential for insecticidal activity. Multi-locus sequence typing analysis (MLST) revealed IBL03679^T^ had a novel sequence type (ST), designated ST610 (PubMLST ID:1185), that did not cluster with any of the seven known *Bacillus cereus senso lato* phylogenetic groupings defined by Guinebretière et al.^2^. Five additional examples of ST610 were subsequently isolated by a different group (NCBI BioProject PRJNA400804; Supplementary Table S1), but were putatively identified in the PubMLST database as *B. cereus*. There are known challenges with identification and differentiation between the *Bacillus cereus senso lato* group species, especially when using the conventional 16S rRNA gene^8^. However, whole-genome sequencing analyses, such as ANIb and dDDH methods, have allowed for more rigorous phylogenetic identification to complement traditional phenotypic characterization approaches^8^. Here, we provide genomic and phenotypic evidence that strain IBL03679^T^ is a *Bacillus* sp. nov. and recommend other ST610s that are currently identified as *B. cereus* be reclassified to *B. silvicola*.

## Materials and methods

### Strain information

Strain IBL03679^T^ was originally isolated from soil sampled from a hardwood forest in central Maryland, USA in 2009 by Blackburn et al., 2014, and bacterial isolation was performed as described^7^. Briefly, 100 mg of dried soil was rehydrated in 10 mL of sterile distilled deionized water (ddH_2_O), vortexed for 1 min, pasteurized for 3 min at 80 °C, and plated onto T3 agar^9^. Colonies were then examined for parasporal crystal formation microscopically and serially subcultured on T3 media to purify. The strain *B. silvicola* IBL03679^T^ was deposited into two repositories: USDA-ARS NRRL (B-65743) and CCUG Sweden (CCUG 78293).

## Colony and cellular morphology

Bacteria were grown on Luria-Bertani 1% agar (LB; Oxoid, Cambridge, UK) at 30 °C for 24 h. Cell morphology was determined using light microscopy (Leica DM LB2 with LAS X software; 1000X/1.25 oil) wet mount and Gram staining (Hardy Diagnostics, Santa Maria, CA, USA). Sporulation and parasporal crystal formation were tested through growth on T3 agar at 30 °C for 72 h and viewed with a phase contrast microscope (Olympus BH-2; 1000X/1.25 oil).

## Physiological, biochemical, and chemotaxonomic testing

To determine the optimal growth temperature for strain IBL03679^T^, a colony from an LB agar plate was resuspended in 1 mL of sterile ddH_2_O, then streaked on the center of each LB agar plate as a single vertical line with a 1 µL calibrated loop. Inoculated agar plates were incubated at 5 °C intervals from 25 to 55 °C, and at 16, 7, and 4 °C. Growth was measured across the inoculation streak at three predetermined points for all temperatures after 24 h and the average was calculated. To find the optimum pH for growth, strain IBL03679^T^ was grown overnight in 50 mL of LB broth at 30 °C and 175 rpm before subculturing 10 µL into flasks with pH increments from 4 to 10. Spectrophotometer (600 nm; NanoDrop™ One^C^, Thermo Scientific, Waltham, MA, USA) readings were measured for each pH flask 5 h after inoculation. Optimal NaCl concentrations for growth were determined using LB agar plates amended with 1% NaCl increments from 0 to 6%. As in the temperature study, bacteria were inoculated and growth measured at 24 h in 30 °C. Anaerobic growth was tested using thioglycolate broth with resazurine (MilliporeSigma, Burlington, MA, USA), and tubes were observed after 48 h at 30 °C for growth. The oxidase test was performed with OxiDrop™ (Hardy Diagnostics), and catalase test with 3% hydrogen peroxide (Hardy Diagnostics) per manufacturer instructions using 24 h cultures grown on LB agar at 30 °C. Hemolytic activity was tested using TSA with 5% blood agar (Remel, San Diego, CA, USA) plates inoculated as in the temperature study and growth measured at 24 h and grown at 30 °C. Motility was tested using 0.3% LB agar swim plates grown at 30 °C for 48 h, and confirmed microscopically with a wet-mount slide using an LB broth culture grown overnight at 30 °C shaking at 200 rpm (Olympus BH-2). *Staphylococcus epidermidis* B-4268 and *Bacillus thuringiensis* HD-1 were used as negative and positive controls, respectively, for the motility and hemolytic assays.

Biochemical testing was performed using an API 50CHB/E test per manufacturer instructions (bioMérieux, Inc., Salt Lake City, UT, USA). Strain IBL03679^T^ was also tested for lecithinase activity on *B. cereus* selective media (Oxoid, Cambridge, UK) amended with chicken egg yolk, casein hydrolysis, and urease activity using methods previously described^6^.

To further characterize strain IBL03679^T^, chemotaxonomic analyses were performed by Leibniz-Institut DSMZ-Deutsche Sammlung von Mikroorganismen und Zellkulturen GmbH Services (DSMZ, Germany) based on methods previously reported^10–13^.

## Whole-genome sequencing and 16S gene amplification

Strain IBL03679^T^ cells were grown in 50 mL of LB broth and shaken at 200 rpm overnight at 30 °C. DNA was extracted using the gram-stain positive protocol for the Puregene Cell Kit (Promega, Madison, WI) per manufacturer instructions and quantified with a QuantiFluor™-ST Fluorometer (Promega). DNA sequencing was conducted using both Illumina MiSeq and Oxford Nanopore (ONP) MinIon instruments. Illumina MiSeq sequencing was performed in-house at the USDA-ARS Invasive Insect Biocontrol and Behavior Laboratory (Beltsville, MD, USA) after library preparation using a KAPA HyperPlus Kit (Roche). ONP MinIon sequencing was performed by the University of Georgia’s Georgia Genomics and Bioinformatics Core (Athens, Georgia, USA). ONP read data were assembled into a draft whole-genome sequence using the Canu program (version 2.2)^14^. Illumina data were aligned to this assembly using Bowtie 2 (version 2.3.4.1)^15^ and the results were used to refine it by polishing with Pilon (version 1.23)^16^. Protein coding genes were predicted using the Prodigal gene finder (version 2.6.3)^17^; tRNA genes were predicted using tRNAscan-SE (version 2.0.12)^18^; and ribosomal RNA features (16S, 23S, and 5S rRNA types) were identified by Barrnap (version 0.9)^19^. Genome quality was quantified using the BUSCO assessment tool (version 6.0.0) with its bacillus_odb12 lineage dataset^20^. The polished assembly and all underlying read data are available from the NCBI WGS and SRA divisions, respectively, under the BioProject accession identifier PRJNA1254167.For 16S gene amplification, DNA was extracted as described above. Polymerase chain reaction (PCR) was performed with TaKaRa Ex Taq (Takara Bio, San Jose, CA, USA) per manufacturer recommendations using primers R16F0 (F: CTGGCTCAGGATTAACGCTGGCGGC) and R16R0 (R: GATACCTTGTTACGACTTAACCCC) to amplify the near-complete 16S rRNA gene (1422 bp)^21^. The amplicon was visualized on an agarose gel (1%, 90 v, 45 min), then purified with ExoSAP-IT Express PCR Product Cleanup kit (Applied Biosystems, Waltham, MA, USA). It was quantified using absorbance with a NanoDrop One^C^ (Thermo Scientific) and fluorescence with a Quantus Fluorometer (Promega) before sending for Sanger sequencing by Eurofins Scientific (Columbia, MD, USA). The sequence was deposited into GenBank with accession PQ123744.

## Phylogeny and genomic analyses

An initial taxonomic comparison for strain IBL03679^T^ was performed with the sequenced 16S rRNA gene amplicon in BLASTn (NCBI). To further classify strain IBL03679^T^, the whole-genome sequence was deposited into the Type Strain Genome Server (TYGS; https://tygs.dsmz.de) for taxonomic classification against the TYGS curated type-strain genome database^1^, and also included other genomic sequences of the ST610 strains identified in the PubMLST database (Supplementary Table S1). The genomic data was compared to closely related species for taxonomic identification via whole-genome sequence comparisons (MASH algorithm)^22^ or the identified 16 S rRNA gene region (RNAmmer and BLAST)^23,24^. Both approaches were subsequently analyzed using the Genome BLAST Distance Phylogeny (GBDP) algorithm and distance formula *d*_*5*_ to determine the closest related type specimens^25^. Phylogenetic trees were inferred with FastME (version 2.1.6.1)^26^ and rooted at the midpoint^27^. They were visualized using the Molecular Ecology Genomic Phylogeny program (MEGA, version 12)^28^. A pairwise comparison using digital DNA-DNA Hybridization (dDDH) was also performed on strain IBL03679^T^ and the five additional ST610 strains with closely related *Bacillus* strains, including within the *Bacillus cereus senso lato* group. Average nucleotide identity (ANI) was measured through JSpeciesWS, an online taxonomic service^29^. Reference genome assemblies were obtained from GenBank for each of the *Bacillus cereus senso lato* group members from the dDDH comparison, as well as for *B. silvicola* IBL03679^T^ and the five other ST610 strains. The UBCG2 pipeline^30^, incorporating the RAxML phylogeny reconstruction program (v.8.2.12)^31^, was used to calculate a phylogenetic tree for these taxa in terms of aligned codons from 81 conserved, single-copy (i.e., “core”) genes.

To annotate the *B. silvicola* IBL03679^T^ genome, the assembly was analyzed using the Bacterial and Viral Bioinformatics Resource Center (BV-BRC; https://www.bv-brc.org/^32^ RASTtk function. Annotated genes were grouped based on predicted function using the BV-BRC tools. Biosynthetic gene clusters (BGC) were identified from the genome assembly of strains *B. silvicola* IBL03679^T^ (Bioproject accession: PRJNA1254167), the five additional *Bacillus* sp. ST610 isolates (Bioproject PRJNA400804), *B. proteolyticus* MCCC 1A00365^T^ and *B. nitratireducens* 4049^T^ using the bacterial antiSMASH program (v7.1.0) with relaxed settings^33^.

## Results and discussion

### Colony and cellular morphology

**Fig. 1:**
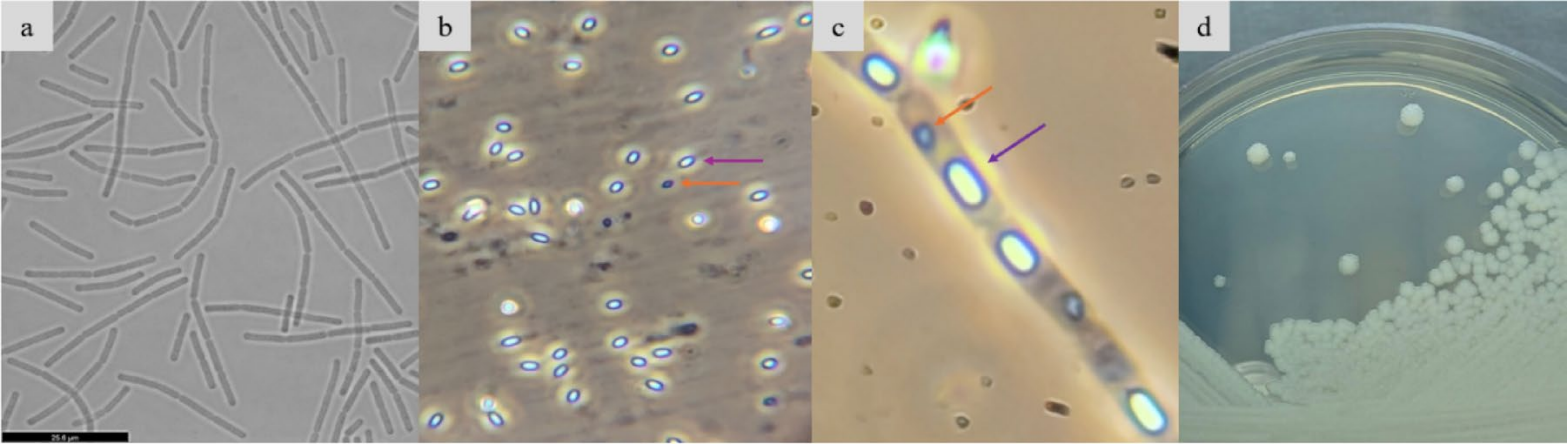
Morphology of *Bacillus silvicola* IBL03679^T^. (**A**) Light microscopy of vegetative cells after 24 h of growth on Luria-Bertani (LB) medium (scale bar 25.6 μm, 1000X objective). (**B**) Phase contrast microscopy of spores (purple arrow) and parasporal crystals (orange arrow) after 6 days of growth on T3 medium (1000X objective, enlarged for visualization), or (**C**) within sporangia after 48 h on T3 agar plates. (**D**) Colonies on LB medium after 24 h of growth at 30 °C.

Bacterial colonies of strain IBL03679^T^ were rough, white, raised, and round with irregular edges (Fig. 1). Vegetative cells were rod-shaped, formed polar chains, and gram-stain positive, with an average of 5.5 μm length and 1.6 μm width. Growth on T3 agar revealed oval non-bulging subterminal endospores and bipyramidal parasporal crystal formation (Fig. 1).

## Physiological and biochemical characterization

All physiological test results are summarized in Table 1 for strain IBL03679^T^ compared to closely related *Bacillus* species in the literature. The optimal growth temperature for strain IBL03679^T^ across all replicates was 30 °C, and no growth was seen on plates grown at 4 °C or ≥ 45 °C. Growth was observed for a pH range of 5–9, with optimum growth at pH 6–7. The optimal NaCl concentration for growth was 0%, with growth observed over a range of 0–4%. Growth was seen throughout the thioglycolate broth tube, indicating facultative anaerobic growth capacity of strain IBL03679^T^. Motility was observed through rapid radial colony expansion on the swim plate after 48 h and via light microscopy (Supplementary Fig S1). Finally, strain IBL03679^T^ lacks cytochrome c oxidase activity, unlike many of the closely related type species, but does produce catalase.

**Table 1:** Physiological characteristics of *B. silvicola* IBL03679^T^ and those of type-specimens of related *Bacillus* species.

Phenotypic test	1	2	3	4	5
Gram stain	+	+	+	+	+
Endospore forming	+	+	+	+	+
Parasporal crystal forming	+	−	−	−	+
Vegetative cell length, width (µm)	4.6–6.1, 1.4–1.9	2.8–3.6, 1.6–1.8	2.7-3.0, 1.0-1.5	3.0–5.0, >1	3.0–5.0, >1
Vegetative cell shape	Rod	Rod	Rod	Rod	Rod
Motility	+	−	−	+ (flagella)	−
Oxygen	Facultative	facultative	facultative	facultative	facultative
Temperature range for growth (optimum), °C	7–40 (30)	10–39 (30)	7–39 (30)	10–45 (opt 30 or 37)	10–45 (opt 30)
pH range (optimum)	4–9 (6–7)	5–10 (8)	5–9 (7)	5-9.5 (opt 6)	5–9.5 (7)
NaCl range (optimum), w/v, %	0–4 (0)	0–9 (0–1)	0–9 (0)	0–7 (opt 0)	0–4 (0)
Casein hydrolysis activity	+	+	+	+	+
Urease activity	+	−	−	+	−
Oxidase/ catalase activity	−/+	+/+	+/+	+/+	+/+

Bacterial strains are as follows: (1) *Bacillus silvicola* IBL03679^T^, (2) *Bacillus proteolyticus* TD42^T^^3^, (3) *Bacillus nitratireducens* 4049^T^^3^, (4) *Bacillus cereus* ATCC 14579^T^^4^, (5) *Bacillus thuringiensis* ATCC 10792^T^^4^. Data for strain 1 obtained from this study, and all others from their respective cited studies. “+” = positive test, “−” = negative test.

Biochemical testing was performed and is summarized in Supplementary Table S2. Distinguishing characteristics for carbohydrate utilization compared to other related *Bacillus* species in the literature is highlighted in Table 2, such as ability of strain IBL03679^T^ to utilize potassium gluconate. Strain IBL03679^T^ also demonstrated lecithinase activity on *B. cereus* selective media (Oxoid, Cambridge, UK). Finally, strain IBL03679^T^ also produced a narrow zone of beta hemolytic activity, similar to that of *Bacillus thuringiensis* HD-1 (Supplementary Fig S1).

**Table 2:** Distinguishing biochemical test results for *Bacillus silvicola* IBL03679^T^ and those of related type-specimens.

API50 CHB/E	1	2	3	4	5
Glycerol	−	−	−	+	−
D-Ribose	+ (weak)	+	+	+	+ (weak)
D-Mannose	−	−	−	−	+
Inositol	−	−	−	+	−
Amygdalin	−	−	−	+ (weak)	−
D-Cellobiose	−	+	−	+	+
D-Saccharose	−	−	−	+	+
L-Fucose	−	−	−	+	−
Potassium Gluconate	+	−	−	−	−

Bacterial strains are as follows: (1) *Bacillus silvicola* IBL03679^T^, (2) *Bacillus proteolyticus* TD42^T^^3^, (3) *Bacillus nitratireducens* 4049^T^^3^, (4) *Bacillus cereus* ATCC 14579^T^^4^, (5) *Bacillus thuringiensis* ATCC 10792^T^^4^. “+” = positive test for acid production, “−” = negative test for acid production.

### Genome assembly and annotation of strain IBL03679^T^

A total of 7,591,961,502 bases spanning 3,324,342 reads was sequenced using the ONP instrument and a total of 1,019,143 Illumina PE250 read pairs containing 472,449,290 bases from the MiSeq system. ONP read data were assembled into a draft whole-genome sequence with a genome size estimate of 6.5 Mb and a corrected error rate of 0.144, which is similar to the 4.09–7.09 Mb size range of previously published *Bacillus cereus senso lato* strains^8^. The polished assembly consisted of 6,909,133 bases assembled into 28 contigs with an N50 of 1,740,814 bp, a minimum contig length of 3,700 bp, and a maximum of 1,896,347 bp. The genome quality was high with an overall BUSCO completeness score of 99.6% (98.3% complete and single-copy, 1.3% complete and duplicated, 0.3% fragmented and 0.1% missing relative to 778 references). The G + C percentage of the overall assembly was 35.49%, which falls within range of 34.5–36.7% previously found in the *Bacillus cereus senso lato* strains^8^ and is similar to the other published ST610s with a range of 35.2–35.3% from the reported published sequences (Supplementary Table S3). A total of 7,101 protein coding genes, 111 tRNA genes, and 42 ribosomal RNA features (14 features apiece for 16S, 23S, and 5S rRNA types) was predicted. Six contigs were flagged as circular, and thus likely corresponded to plasmids: tig00000007_pilon (184,192 bp), tig00000008_pilon (114,464 bp), tig00000023_pilon (541,737 bp), tig00000025_pilon (153,138 bp), tig00000030_pilon (153,207 bp), and tig00000033_pilon (12,731 bp). Genomic annotation of strain IBL03679^T^ using BC-BRC (genome ID: 1386.5282) further identified 2,297 hypothetical protein coding genes within the genome, as well as 65 transport associated genes, 65 genes associated with sporulation, 55 genes associated with antimicrobial resistance, and 4 genes associated with metal resistance.

### Phylogenetic characterization

An initial taxonomic comparison for strain IBL03679^T^ was performed with the sequenced 16S rRNA gene amplicon using BLASTn. Due to high sequence similarity of 16S rRNA genes within the *Bacillus cereus senso lato* group^8^, it was not possible to differentiate strain IBL03679^T^ using this sequence alone. Most type species from that group matched 100% with the strain IBL03679^T^ sequence.

**Fig. 2:**
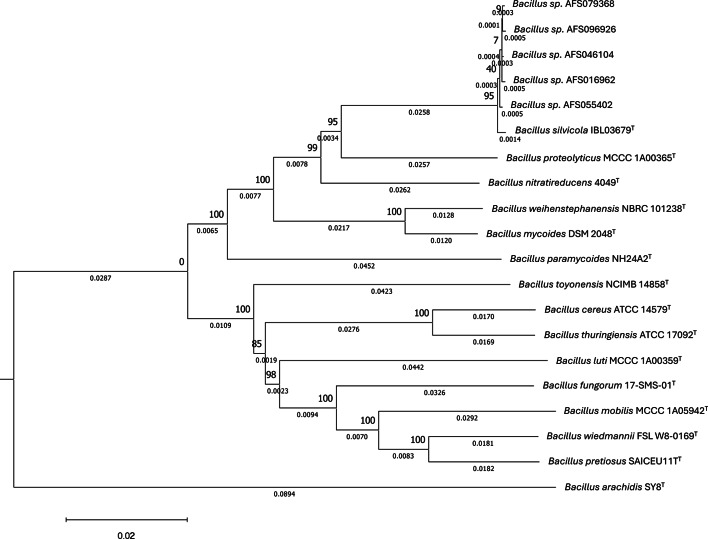
Whole-genome phylogram of *B. silvicola* IBL03679^T^ with additional ST610 strains and other related type-species. Tree inferred with FastME using GBDP distances calculated from genome sequences. The branch lengths are scaled in terms of GBDP distance formula *d*_*5*_. The numbers above branches are GBDP pseudo-bootstrap support values > 60% from 100 replications, with an average branch support of 79.2%. The tree is midpoint-rooted.

**Fig. 3:**
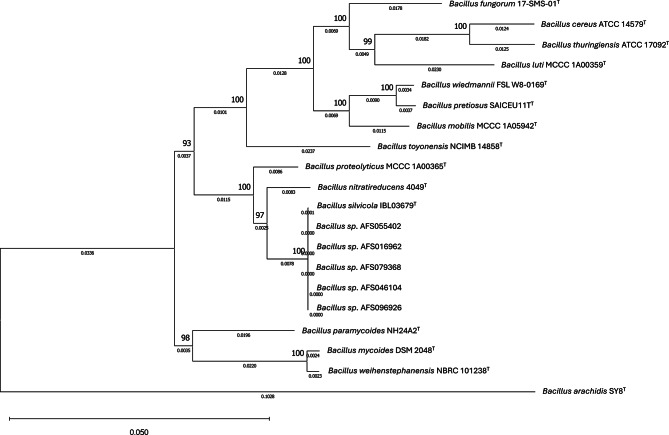
Phylogram of *Bacillus cereus senso lato* members using conserved, single-copy genes. Branch lengths are shown as decimal values, and node labels represent bootstrap support (100 replicates) obtained from 81 core genes shared among the reference genomes. The tree is midpoint-rooted.

To further classify *B. silvicola* IBL03679^T^ and the other ST610 *Bacillus* sp. strains identified in the PubMLST database (Supplementary Table S1), the whole-genome sequences were deposited into the Type Strain Genome Server (TYGS; https://tygs.dsmz.de) for taxonomic classification against the TYGS curated type-strain genome database^1^. In the 16S rRNA gene phylogenetic tree, there was a low average branch support (46.5%) with small branch lengths and delta statistic of 0.247, which is typical for *Bacillus cereus senso lato* group due to their highly similar 16S rRNA gene sequences (Supplementary Fig S1). Even so, the five additional ST610 *Bacillus* strains all clustered with strain IBL03679^T^ within the same clade and exhibited short branch lengths. Alternatively, the whole-genome sequences provided a higher average branch support of 79.2% and lower delta statistic of 0.141 (Fig. 2), and also supported relatedness with high node confidence and short branch lengths within the clade between all ST610 strains. The dDDH percent similarity of strain IBL03679^T^ and the other ST610 *Bacillus* sp. strains to the tested *Bacillus* type-strains ranged from 27.2 to 59.5%, which is well below the common 70% threshold for species delineation (*d*_*4*_ algorithm; Supplementary Table S4). All six ST610 strains had highly similar genomes, with 99.1–99.6% similarity between strains and 97.5–98.2% similarity with strain IBL03679^T^ (*d*_*4*_ algorithm), demonstrating they are conspecifics. Based on the dDDH and whole-genome phylogeny results, the closest relative to strain IBL03679^T^ and the other ST610 *Bacillus* sp. strains was identified as *Bacillus proteolyticus* MCCC 1A00365^T^, with a dDDH range of 59.5–59.9%, followed by *B. nitratireductans* 4049^T^ and *B. mycoides* DSM 2048^T^. ANIb (ANI based on BLAST) results showed > 99% match between all ST610s and strain IBL03679^T^, whereas the closest match for each of them fell below the conventional 95–96% threshold and was also to *B. proteolyticus* MCCC 1A00365^T^, then *B. nitratireductans* 4049^T^ and *B. mycoides* DSM 2048^T^ (Supplementary Table S5). Although quite similar overall to the phylogeny shown in Fig. 2, the tree presented in Fig. 3, based on conserved, single-copy genic content, indicates that *B. silvicola* is more closely related to *B. nitratireducens* 4049^T^ than to *B. proteolyticus* MCCC 1A00365^T^. Branch lengths among these three taxa are quite small, however, indicating that the genetic determinants that effected speciation among these bacteria mainly accumulated in genomic features distinct from core genes.

### Biosynthetic gene cluster analysis

Biosynthetic gene cluster (BGC) identification within strain IBL03679^T^ and the other ST610 strains revealed similar clusters within each genome. Strain IBL03679^T^ had 15 clusters putatively identified (Supplementary Table S6), while the other ST610 strains had a range of 9–12 clusters identified, yet all but four were unnamed. Of those, only two had high similarity matches (> 75%) to known clusters in the database, the siderophores bacillibactin and petrobactin, and one moderate match (50–75%), the lasso peptide paeninodin. All six strains contained these three BGC at these match levels, showing a possible species-level trait in antimicrobial production potential. The BGC were also determined for the two closest related species, *B. proteolyticus* MCCC 1A00365^T^ and *B. nitratireducens* 4049^T^, and the same three BGC were found, suggesting the potential activity is conserved within the clade. Interestingly, the *B. nitratireducens* 4049^T^ had a high similarity match to the paeninodin lasso peptide, suggesting a divergence of this trait in the clade containing *B. proteolyticus* and the *B. silvicola* strains.

### Chemotaxonomic identifiers

To further characterize strain IBL03679^T^, chemotaxonomic analyses were performed. The fatty acid methyl esters (FAMEs) detected are provided in Supplementary Table S7. The dominant detected FAMEs were C_16:0_, iso-C_15:0_, and iso-C_17:0_, which comprised 44.6% of the total and were similar to the most dominant FAMEs previously reported for the other listed type species^3,4^. Smaller amounts of iso-C_12:0_, C_12:0_, C_15:0_, iso-C_18:0_, and C_18:1_ ω9c were also detected, unlike most other represented type-species. The polar lipid profile identified for strain IBL03679^T^ was primarily diphosphatidylglycerol (DPG), phosphatidylethanolamine (PE), and phosphatidylglyercol (PG), with lesser amounts of phospholipids, glycolipids, aminophospholipids, and lipids detected (Supplementary Fig S3). Menaquinone-7 (MK-7) was the sole respiratory quinone identified. The peptidoglycan type was found to be A1γ meso-Dpm – direct (A31).

### Taxonomic conclusion

Based upon the physiological, biochemical, and chemotaxonomic tests, as well as genomic analysis and phylogenetic comparisons, it can be concluded that strain IBL03679^T^ is a novel species belonging to the genus *Bacillus*, with the proposed species name of *Bacillus silvicola*. Further, this information suggests the five additional ST610 strains, originally identified as *Bacillus cereus*, instead belong to the same novel *Bacillus* species as strain IBL03679^T^.

Description of *Bacillus silvicola* sp. nov.

*Bacillus silvicola* (sil.vi’co.la. L. fem. n. *silva*, a forest; L. suff. -*cola*, from L. masc. or fem. *incola*, inhabitant, dweller; N.L. masc. n. *silvicola*, an inhabitant of the forest, from the place of isolation). Bacteria are gram-stain positive, rod-shaped cells with a 4.6–6.1 μm length and 1.4–1.9 μm width, and are motile. They can produce endospores and parasporal protein crystals. Colonies are rough, white, raised, and round with irregular edges. Bacterial growth was observed for a temperature range of 15–40 °C, NaCl range of 0–4%, and pH range of 5–9, with optimal growth at 30 °C, 0% NaCl, and pH 6–7. Strain IBL03679^T^ is positive for catalase production, does not have cytochrome C oxidase activity, has beta-hemolytic activity, can hydrolyze casein, and can produce lecithinase. Distinguishing biochemical test results for strain IBL03679^T^ include an inability to metabolize D-cellobiose and D-saccharose, and positive acid production with potassium gluconate. Strain IBL03679^T^ is also positive for esculin hydrolysis, and produces acid with D-glucose, D-fructose, N-acetylglucosamine, arbutin, salicin, D-maltose, D-trehalose, starch, glycogen, and (weakly) D-ribose. The major fatty acids identified are C_16:0_, iso-C_15:0_, and iso-C_17:0_, and major polar lipids identified are diphosphatidylglycerol (DPG), phosphatidylethanolamine (PE), and phosphatidylglyercol (PG). The respiratory quinone is menaquinone-7 (MK-7), and the peptidoglycan type is A1γ meso-Dpm – direct (A31). Strain IBL03679^T^ (= NRRL B-65743^T^, = CCUG 78293^T^), the type strain for *Bacillus silvicola* sp. nov., was isolated from Maryland hardwood forest soil. The genomic G + C content of the sequenced strains ranges from 35.16 to 35.54% with genome assembly sizes ranging 5.7–6.9 Mbp. The complete genome assembly for strain IBL03679^T^ can be found in GenBank under BioProject accession number PRJNA1254167, and the 16S rRNA gene sequence at accession number PQ123744.

## Supplementary Information

Below is the link to the electronic supplementary material.

Supplementary Material 1

## Data Availability

The whole-genome assembly of strain IBL03679^T^ can be found at the NCBI BioProject data repository with accession PRJNA1254167. The 16S rRNA gene sequence data for strain IBL03679^T^ is available in NCBI with accession PQ123744. The strain *B. silvicola* IBL03679^T^ was deposited into two locations: USDA-ARS NRRL (B-65743; https://apps.ars.usda.gov/nrrl) and CCUG Sweden (CCUG 78293; https://www.ccug.se/).

## Abbreviations

dDDH: Digital DNA-DNA hybridization
ANIb: Average nucleotide identity
LB: Luria-Bertani media
PCR: Polymerase chain reaction
MLST: Multi-locus sequence typing
ST: Sequence type
