# Supplementary material for: Bacillus silvicola sp. nov., a new species within the Bacillus cereus group isolated from hardwood forest soil in Maryland, USA
Source: Sci Rep. 2026 May 13;16:21915. doi: 10.1038/s41598-026-52504-9 (PMC13365238; doi:10.1038/s41598-026-52504-9)
Supplement: Supplementary file 1 — Supplementary Material 1 [file 41598_2026_52504_MOESM1_ESM.docx]

**Supplementary Materials**

***Bacillus silvicola* sp. nov., a new species within the *Bacillus cereus* group isolated from hardwood forest soil in Maryland, USA**

**Holly P. Bartholomew*, Michael E. Sparks, Daniel Kuhar, Ashaki Mitchell, Robert R. Farrar Jr., Dawn E. Gundersen-Rindal, Michael B. Blackburn**

Invasive Insect Biocontrol and Behavior Laboratory, USDA-ARS, Henry A. Wallace Beltsville Agricultural Research Center, Beltsville, MD, 20705, USA

*Corresponding author: holly.bartholomew@usda.gov

**Supplementary Table S1.** Multi-locus sequence typing (MLST) identification reveals closely related strains in group ST610^a^

| **Strain information** | | | | **MLST** | | | | | | | |
| --- | --- | --- | --- | --- | --- | --- | --- | --- | --- | --- | --- |
| **PubMLST Id** | **Strain** | **Accession numbers and aliases** | **Year** | **glp** | **gmk** | **ilv** | **pta** | **pur** | **pyc** | **tpi** | **ST** |
| 1185 | IBL03679^T^ | IBL 3679; PRJNA1254167 | 2009 | 146 | 85 | 157 | 144 | 143 | 130 | 115 | 610 |
| 3518 | AFS016962 | NTUE01; PRJNA400804; GCA_002561265.1 | 2014 | 146 | 85 | 157 | 144 | 143 | 130 | 115 | 610 |
| 3629 | AFS055402 | NUGR01; PRJNA400804; GCA_002574955.1 | 2014 | 146 | 85 | 157 | 144 | 143 | 130 | 115 | 610 |
| 3670 | AFS046104 | NUJQ01; PRJNA400804; GCA_002576065.1 | 2014 | 146 | 85 | 157 | 144 | 143 | 130 | 115 | 610 |
| 3819 | AFS079368 | NUXX01; PRJNA400804; GCA_002565695.1 | 2014 | 146 | 85 | 157 | 144 | 143 | 130 | 115 | 610 |
| 3939 | AFS096926 | NVLJ01; PRJNA400804; GCA_002550375.1 | 2014 | 146 | 85 | 157 | 144 | 143 | 130 | 115 | 610 |

^a^All strains from the USA. All 2014 strains identified as variety serovar ‘cereus’. Information from PubMLST.

**Supplementary Table S2.** Full biochemical test results for *Bacillus silvicola* IBL03679^T^ and those of related type-specimens^a^

| **API50 CHB/E** | **1** | **2** | **3** | **4** | **5** |
| --- | --- | --- | --- | --- | --- |
| Glycerol | − | − | − | + | − |
| Erythritol | − | − | − | − | − |
| D-Arabinose | − | − | − | − | − |
| L-Arabinose | − | − | − | − | − |
| D-Ribose | + (weak) | + | + | + | + (weak) |
| D-Xylose | − | − | − | − | − |
| L-Xylose | − | − | − | − | − |
| D-Adonitol | − | − | − | − | − |
| Methyl-Beta-D-xylopyranoside | − | − | − | − | − |
| D-Galactose | − | − | − | − | − |
| D-Glucose | + | + | + | + | + |
| D-Fructose | + | + | + | + | + |
| D-Mannose | − | − | − | − | + |
| L-Sorbose | − | − | − | − | − |
| L-Rhamnose | − | − | − | − | − |
| Dulcitol | − | − | − | − | − |
| Inositol | − | − | − | + | − |
| D-Mannitol | − | − | − | − | − |
| D-Sorbitol | − | − | − | − | − |
| Methyl-Alpha-D-Mannopyranoside | − | − | − | − | − |
| Methyl-Alpha-D-Glucopyranoside | − | − | − | − | − |
| N-Acetylglucosamine | + | + | + | + | + |
| Amygdalin | − | − | − | + (weak) | − |
| Arbutin | + | + | + | + | + |
| Esculin / Ferric Citrate | + | + | + | + | + |
| Salicin | + | + | + | + | + |
| D-Cellobiose | − | + | − | + | + |
| D-Maltose | + | + | + | + | + |
| D-Lactose (bovine origin) | − | − | − | − | − |
| D-Melibiose | − | − | − | − | − |
| D-Saccharose (sucrose) | − | − | − | + | + |
| D-Trehalose | + | + | + | + | + |
| Inulin | − | − | − | − | − |
| D-Melezitose | − | − | − | − | − |
| D-Raffinose | − | − | − | − | − |
| Starch (amidon) | + | + | + | + | + |
| Glycogen | + | + | + | + | + |
| Xylitol | − | − | − | − | − |
| Gentibiose | − | − | − | − | − |
| D-Turanose | − | − | − | − | − |
| D-Lyxose | − | − | − | − | − |
| D-Tagatose | − | − | − | − | − |
| D-Fucose | − | − | − | − | − |
| L-Fucose | − | − | − | + | − |
| D-Arabitol | − | − | − | − | − |
| L-Arabitol | − | − | − | − | − |
| Potassium Gluconate | + | − | − | + | − |
| Potassium 2-Ketogluconate | − | − | − | − | − |
| Potassium 5-Ketogluconate | − | − | − | − | − |

^a^Strains are as follows: 1) *Bacillus silvicola* IBL03679^T^, 2) *Bacillus proteolyticus* TD42^T^ [3], 3) *Bacillus nitratireducens* 4049^T^ [3], 4) *Bacillus cereus* ATCC 14579^T^ [4], 5) *Bacillus thuringiensis* ATCC 10792^T^ [4]. Data for strain 1 obtained from this study, and all others from their respective identification studies. “+” = positive test for acid production, “−” = negative test for acid production.

**Supplementary Table S3.** Genome size and GC percent of strain IBL03679^T^, *Bacillus* ST610s, and other *Bacillus* type-strains

| **Strain** | **GC (%)** | **Genome size (bp)** |
| --- | --- | --- |
| *Bacillus thuringiensis* ATCC 10792^T^ | 34.68 | 6,260,142 |
| *Bacillus fungorum* 17-SMS-01^T^ | 34.99 | 5,651,163 |
| *Bacillus mycoides* ATCC 6462^T^ | 35.08 | 5,561,906 |
| *Bacillus nitratireducens* 4049^T^ | 35.13 | 5,933,421 |
| *Bacillus proteolyticus* TD42^T^ | 35.15 | 5,859,329 |
| *Bacillus sp*. AFS096926^a^ | 35.16 | 5,943,482 |
| *Bacillus sp.* AFS079368^a^ | 35.17 | 5,839,159 |
| *Bacillus weihenstephanensis* NBRC 101238^T^ | 35.18 | 5,591,859 |
| *Bacillus toyonensis* BCT-7112^T^ | 35.19 | 5,787,787 |
| *Bacillus sp.* AFS016962^a^ | 35.19 | 5,831,025 |
| *Bacillus paramycoides* NH24A2^T^ | 35.21 | 5,453,107 |
| *Bacillus sp*. AFS046104^a^ | 35.21 | 5,729,173 |
| *Bacillus sp.* AFS055402^a^ | 35.23 | 5,729,747 |
| *Bacillus cereus* ATCC 14579^T^ | 35.29 | 5,431,377 |
| *Bacillus mobilis* 0711P9-1^T^ | 35.3 | 5,660,141 |
| *Bacillus pretiosus* SAICEU11T^T^ | 35.32 | 5,385,342 |
| *Bacillus wiedmannii* FSL W8-0169^T^ | 35.46 | 5,585,953 |
| *Bacillus silvicola* IBL03679^T^ | 35.49 | 6,908,876 |
| *Bacillus arachidis* SY8^T^ | 35.54 | 5,218,944 |
| *Bacillus luti* TD41^T^ | 35.71 | 5,315,231 |

^a^ST610 strains identified by PubMLST database.

**Supplementary Table S4.** dDDH-based whole-genome pairwise comparisons of *B. silvicola* IBL03679^T^, other *Bacillus* ST610 strains, and *Bacillus* species type-strains^a^

|  | **dDDH (*d4*, %), [C.I. (*d4*, %)], G+C content difference (%)** | | | | | |
| --- | --- | --- | --- | --- | --- | --- |
| ***Bacillus* subject strain** | (1) | (2) | (3) | (4) | (5) | (6) |
| **(1)** | * |  |  |  |  |  |
| **(2)** | 98.2, [97.4 - 98.8], 0.26 | * |  |  |  |  |
| **(3)** | 98, [97.1 - 98.6], 0.28 | 99.3, [98.8 - 99.5], 0.01 | * |  |  |  |
| **(4)** | 97.7, [96.7 - 98.4], 0.3 | 99.1, [98.5 - 99.4], 0.04 | 99.4, [99.1 - 99.6], 0.02 | * |  |  |
| **(5)** | 97.6, [96.6 - 98.3], 0.32 | 99.1, [98.6 - 99.4], 0.05 | 99.6, [99.4 - 99.8], 0.04 | 99.3, [98.9 - 99.6], 0.02 | * |  |
| **(6)** | 97.5, [96.5 - 98.2], 0.33 | 99.1, [98.6 - 99.4], 0.06 | 99.3, [98.9 - 99.6], 0.05 | 99.2, [98.8 - 99.5], 0.03 | 99.4, [99.0 - 99.6], 0.01 | * |
| ***Bacillus proteolyticus* TD42^T^ (GCF 001884065)** | 59.5, [56.7 - 62.3], 0.34 | 59.8, [57.0 - 62.6], 0.08 | 59.9, [57.1 - 62.7], 0.06 | 59.7, [56.9 - 62.5], 0.04 | 59.7, [56.8 - 62.4], 0.02 | 59.6, [56.8 - 62.4], 0.01 |
| ***Bacillus nitratireducens* 4049^T^ (GCF 001884135)** | 57.5, [54.7 - 60.2], 0.18 | 57.4, [54.6 - 60.1], 0.08 | 57.4, [54.6 - 60.1], 0.09 | 57.4, [54.6 - 60.2], 0.12 | 57.4, [54.6 - 60.1], 0.13 | 57.4, [54.6 - 60.1], 0.14 |
| ***Bacillus mycoides* ATCC 6462^T^ (GCA 000003925)** | 50.2, [47.6 - 52.8], 0.29 | 50, [47.4 - 52.6], 0.02 | 50, [47.3 - 52.6], 0.01 | 50, [47.3 - 52.6], 0.02 | 50, [47.3 - 52.6], 0.03 | 49.9, [47.3 - 52.6], 0.04 |
| ***Bacillus weihenstephanensis* NBRC 101238 (GCA 000513155)** | 49.2, [46.6 - 51.8], 0.29 | 49.3, [46.7 - 52.0], 0.02 | 49.3, [46.7 - 52.0], 0.01 | 49.3, [46.7 - 51.9], 0.01 | 49.3, [46.7 - 51.9], 0.03 | 49.3, [46.7 - 51.9], 0.04 |
| ***Bacillus paramycoides* NH24A2^T^ (GCF 001884235)** | 44.2, [41.6 - 46.7], 0.28 | 44, [41.5 - 46.5], 0.02 | 44, [41.5 - 46.6], 0 | 44.1, [41.5 - 46.6], 0.02 | 43.9, [41.4 - 46.5], 0.04 | 43.8, [41.3 - 46.4], 0.05 |
| ***Bacillus toyonensis* BCT-7112^T^ (GCA 000496285)** | 42, [39.5 - 44.6], 0.06 | 41.7, [39.2 - 44.3], 0.33 | 41.7, [39.2 - 44.3], 0.34 | 41.8, [39.3 - 44.3], 0.36 | 41.7, [39.2 - 44.3], 0.38 | 41.7, [39.2 - 44.3], 0.39 |
| ***Bacillus pretiosus* SAICEU11T^T^ (GCA 025916425)** | 41.2, [38.7 - 43.7], 0.17 | 41, [38.5 - 43.6], 0.09 | 41.1, [38.6 - 43.6], 0.11 | 41.1, [38.6 - 43.6], 0.13 | 41, [38.5 - 43.6], 0.15 | 41, [38.6 - 43.6], 0.16 |
| ***Bacillus wiedmannii* FSL W8-0169^T^ (GCF 001583695)** | 41, [38.5 - 43.6], 0.3 | 40.9, [38.4 - 43.5], 0.04 | 40.9, [38.5 - 43.5], 0.02 | 41, [38.5 - 43.5], 0 | 40.9, [38.5 - 43.5], 0.02 | 40.9, [38.5 - 43.5], 0.03 |
| ***Bacillus mobilis* 0711P9-1^T^ (GCF 001884045)** | 40.6, [38.1 - 43.1], 0.19 | 40.5, [38.0 - 43.0], 0.24 | 40.4, [37.9 - 43.0], 0.23 | 40.5, [38.0 - 43.0], 0.2 | 40.4, [38.0 - 43.0], 0.18 | 40.5, [38.0 - 43.0], 0.18 |
| ***Bacillus fungorum* 17-SMS-01^T^ (GCA 002746455)** | 40.4, [37.9 - 42.9], 0.5 | 40.3, [37.9 - 42.9], 0.07 | 40.4, [37.9 - 42.9], 0.09 | 40.3, [37.9 - 42.9], 0.11 | 40.3, [37.8 - 42.9], 0.13 | 40.3, [37.8 - 42.8], 0.14 |
| ***Bacillus luti* TD41^T^ (GCF 001884105)** | 40.3, [37.8 - 42.9], 0.04 | 40.1, [37.6 - 42.6], 0.23 | 40.1, [37.6 - 42.7], 0.24 | 40.1, [37.6 - 42.7], 0.26 | 40.1, [37.6 - 42.7], 0.28 | 40.1, [37.6 - 42.6], 0.29 |
| ***Bacillus cereus* ATCC 14579^T^ (GCA 045287585.1)** | 39.7, [37.2 - 42.2], 0.22 | 39.5, [37.0 - 42.0], 0.05 | 39.5, [37.0 - 42.0], 0.06 | 39.5, [37.0 - 42.0], 0.08 | 39.5, [37.0 - 42.0], 0.1 | 39.5, [37.0 - 42.0], 0.11 |
| ***Bacillus thuringiensis* ATCC 10792^T^ (GCA 000161615)** | 39.5, [37.0 - 42.0], 0.67 | 39.5, [37.0 - 42.0], 0.41 | 39.4, [36.9 - 41.9], 0.39 | 39.5, [37.0 - 42.0], 0.37 | 39.4, [36.9 - 41.9], 0.35 | 39.4, [36.9 - 41.9], 0.34 |
| ***Bacillus arachidis* SY8^T^ (GCA 017498775)** | 27.6, [25.2 - 30.1], 0.14 | 27.2, [24.9 - 29.7], 0.12 | 27.2, [24.8 - 29.7], 0.13 | 27.3, [24.9 - 29.8], 0.16 | 27.3, [24.9 - 29.7], 0.17 | 27.3, [24.9 - 29.8], 0.18 |

^a^Numbered strains are (1) *B. silvicola* IBL03679^T^, (2) *B. cereus* AFS055402, (3) *B. cereus* AFS046104, (4) *B. cereus* AFS016962, (5) *B. cereus* AFS079368, (6) *B. cereus* AFS096926. Strains 1-6 identified as ST610 in PubMLST database.^T^ = type-strain. C.I. = confidence interval. *d4* denotes the reference the genome BLAST distance phylogeny (GBDP) formula used by the TYGS system.

**Supplementary Table S5.** ANIb-based whole-genome comparisons of *Bacillus* type-specimens with classified ST610s^a^

|  | ANIb (%) [Aligned (%)] | | | | | |
| --- | --- | --- | --- | --- | --- | --- |
| Bacillus subject strain | (1) | (2) | (3) | (4) | (5) | (6) |
| (1) | * |  |  |  |  |  |
| (2) | 99.78 [96.40] | * |  |  |  |  |
| (3) | 99.54 [94.16] | 99.65 [94.43] | * |  |  |  |
| (4) | 99.59 [95.12] | 99.62 [95.09] | 99.79 [96.61] | * |  |  |
| (5) | 99.59 [94.84] | 99.65 [95.11] | 99.83 [96.83] | 99.77 [96.22] | * |  |
| (6) | 99.75 [95.90] | 99.80 [96.54] | 99.85 [97.07] | 99.83 [96.63] | 99.92 [97.01] | * |
| *Bacillus proteolyticus* TD42^T^ | 94.17 [79.23] | 94.28 [78.89] | 94.26 [79.37] | 94.24 [79.07] | 94.27 [79.23] | 94.20 [78.76] |
| *Bacillus nitratireducens* 4049^T^ | 93.80 [79.72] | 93.80 [80.10] | 93.81 [80.16] | 93.79 [80.00] | 93.84 [80.19] | 93.77 [79.96] |
| *Bacillus mycoides* ATCC 6462^T^ | 92.27 [76.43] | 92.30 [76.27] | 92.25 [76.37] | 92.22 [76.16] | 92.26 [76.33] | 92.23 [76.02] |
| *Bacillus paramycoides* NH24A2^T^ | 90.80 [75.72] | 90.83 [75.32] | 90.78 [76.04] | 90.84 [75.62] | 90.84 [75.75] | 90.78 [75.13] |
| *Bacillus toyonensis* BCT-7112^T^ | 90.33 [78.29] | 90.44 [77.37] | 90.40 [77.53] | 90.42 [77.54] | 90.43 [77.42] | 90.45 [77.45] |
| *Bacillus wiedmannii* FSL W8-0169^T^ | 89.92 [75.05] | 89.95 [75.29] | 89.95 [75.30] | 89.92 [75.30] | 89.95 [75.28] | 89.94 [75.27] |
| *Bacillus pretiosus* SAICEU11T^T^ | 89.92 [73.77] | 89.95 [73.80] | 89.95 [73.90] | 89.95 [73.82] | 89.95 [73.89] | 89.98 [73.63] |
| *Bacillus luti* TD41^T^ | 89.80 [77.21] | 89.80 [77.64] | 89.80 [77.61] | 89.79 [77.55] | 89.80 [77.59] | 89.80 [77.52] |
| *Bacillus mobilis* 0711P9-1^T^ | 89.40 [71.25] | 89.43 [71.54] | 89.38 [71.62] | 89.44 [71.43] | 89.43 [71.49] | 89.44 [71.42] |
| *Bacillus paramobilis* BML-BC017^T^ | 89.40 [70.73] | 89.45 [70.77] | 89.39 [70.99] | 89.44 [70.78] | 89.42 [70.98] | 89.42 [70.74] |
| *Bacillus cereus* ATCC 14579^T^ | 89.37 [74.06] | 89.47 [73.70] | 89.44 [73.74] | 89.43 [73.62] | 89.44 [73.68] | 89.41 [73.67] |
| *Bacillus thuringiensis* ATCC 10792^T^ | 88.88 [65.77] | 89.11 [64.84] | 88.97 [65.35] | 89.02 [65.04] | 89.07 [65.04] | 89.02 [64.92] |
| *Bacillus fungorum* 17-SMS-01^T^ | 89.41 [68.19] | 89.56 [67.10] | 89.53 [67.31] | 89.54 [67.23] | 89.55 [67.32] | 89.57 [67.03] |

^a^Numbered strains are (1) *B. silvicola* IBL03679^T^, (2) *B. cereus* AFS055402, (3) *B. cereus* AFS096926, (4) *B. cereus* AFS016962, (5) *B. cereus* AFS079368, (6) *B. cereus* AFS046104. Strains 2-6 identified as ST610 and labeled *B. cereus* in PubMLST database.^T^ = type-strain

**Supplementary Table S6.** Full biosynthetic gene cluster identification list for *B silvicola* IBL03679^T^

| **Type** | **Most similar known cluster** | **Similarity Confidence** |
| --- | --- | --- |
| **NI-siderophore** | petrobactin | High (> 75%) |
| **NRP-metallophore,NRPS** | bacillibactin | High (> 75%) |
| **lassopeptide** | paeninodin | Medium (50-75%) |
| **betalactone** | fengycin | Low (< 50%) |
| **azole-containing-RiPP** | ̶ | ̶ |
| **deazapurine** | ̶ | ̶ |
| **lanthipeptide-class-ii** | ̶ | ̶ |
| **lanthipeptide-class-ii** | ̶ | ̶ |
| **NAPAA** | ̶ | ̶ |
| **ranthipeptide** | ̶ | ̶ |
| **RiPP-like** | ̶ | ̶ |
| **RiPP-like** | ̶ | ̶ |
| **RiPP-like** | ̶ | ̶ |
| **terpene** | ̶ | ̶ |
| **terpene-precursor** | ̶ | ̶ |

**Supplementary Table S7.** Cellular fatty acid compositions (%) for *B. silvicola* IBL03679^T^ and related *Bacillus* strains^a^

| **Fatty acid** | **1** | **2** | **3** | **4** | **5** |
| --- | --- | --- | --- | --- | --- |
| iso-C_12 : 0_ | 2.5 | 2.3 | 2.2 | 1.9 | 1.5 |
| C_12 : 0_ | 1.5 | 3.3 | 4.7 | 1.4 | 1 |
| iso-C_13 : 0_ | **9.9** | **8.8** | **8.5** | **20.3** | **18.5** |
| anteiso-C_13 : 0_ | 2.9 | 2.9 | 2.4 | 4 | 2.8 |
| iso-C_14 : 0_ | **8.5** | 2.7 | 1.8 | 4.8 | **5.2** |
| C_14 : 0_ | 3.2 | **6.5** | **7.7** | 4.1 | 4.1 |
| iso-C_15 : 0_ | **15.1** | **8.3** | **5.5** | **20.2** | **21.8** |
| anteiso-C_15 : 0_ | **6.6** | 3.9 | 2.4 | **6.5** | **5.3** |
| C_15 : 0_ | TR | ND | ND | ND | ND |
| iso-C_15 : 1_G | ND | TR | TR | ND | ND |
| C_16 : 1_ω7*c* alcohol | TR^b^ | TR | TR | ND | ND |
| Summed feature 2 | TR^c^ | ND | TR | ND | ND |
| iso-C_16 : 0_ | **8.4** | 3.4 | 3.2 | 3.3 | 3.7 |
| iso-C_16 : 1_ ω5 | ND | ND | ND | TR | 1.3 |
| C_16 : 1_ ω6c | ND | ND | ND | **5.9** | **7.5** |
| C_16 : 1_ ω11c | TR | 2.1 | 2.4 | TR | TR |
| Summed feature 3 | 2.7^d^ | **5.5** | **5.6** | ND | ND |
| C_16 : 0_ | **16.2** | **33.3** | **30.5** | **12.5** | **10.3** |
| iso-C_17 : 1_ω11 | ND | ND | ND | 2.7 | 3.2 |
| iso-C_17 : 1_ω6 | ND | ND | ND | 1 | 2.6 |
| iso-C_17 : 1_ω10*c* | TR^e^ | 1.6 | 1.8 | ND | ND |
| iso-C_17 : 1_ω5*c* | TR^f^ | TR | TR | ND | ND |
| anteiso-C_17 : 1_ A | ND | ND | TR | ND | ND |
| iso-C_17 : 0_ | **13.3** | 4.3 | 2.5 | **6.7** | **6.9** |
| anteiso-C_17 : 0_ | 3.2 | 1.5 | 1.1 | 1.5 | 1.1 |
| anteiso-C_17 : 1_ ω6 | ND | ND | ND | TR | TR |
| C_17 : 0_ | TR | ND | ND | ND | ND |
| iso-C_18 : 0_ | TR | ND | ND | ND | ND |
| C_18 : 1_ ω9c | TR | 1 | 1.6 | TR | TR |
| C_18 : 0_ | 1.9 | **5.3** | **9.2** | TR | TR |
| Summed feature 8 | ND | 2.1 | 4.1 | ND | ND |

^a^Bacterial strains are as follows: 1) *Bacillus silvicola* IBL03679^T^, 2) *Bacillus proteolyticus* TD42^T^ [3], 3) *Bacillus nitratireducens* 4049^T^ [3], 4) *Bacillus cereus* ATCC 14579^T^ [4], 5) *Bacillus thuringiensis* ATCC 10792^T^ [4]; ND = not detected or no data, TR = Trace amount (<1%), bold > 5%. Data for strain 1 obtained from this study, and all others from their respective cited studies.

^b^identified as iso-C_16 : 1_ ω10c

^c^Summed feature 2 listed as iso-C_16 : 1_ I and/or C_14 :0_ 3-OH, identified as iso-C_16 : 1_ ω5c

^d^Summed feature 3 listed as C_16 : 1_ ω6c and/or C_16 : 1_ ω7c, identified as C_16 : 1_ ω6c

^e^identified as iso-C_17 : 1_ ω11c

^f^identified as iso-C_17 : 1_ ω6c


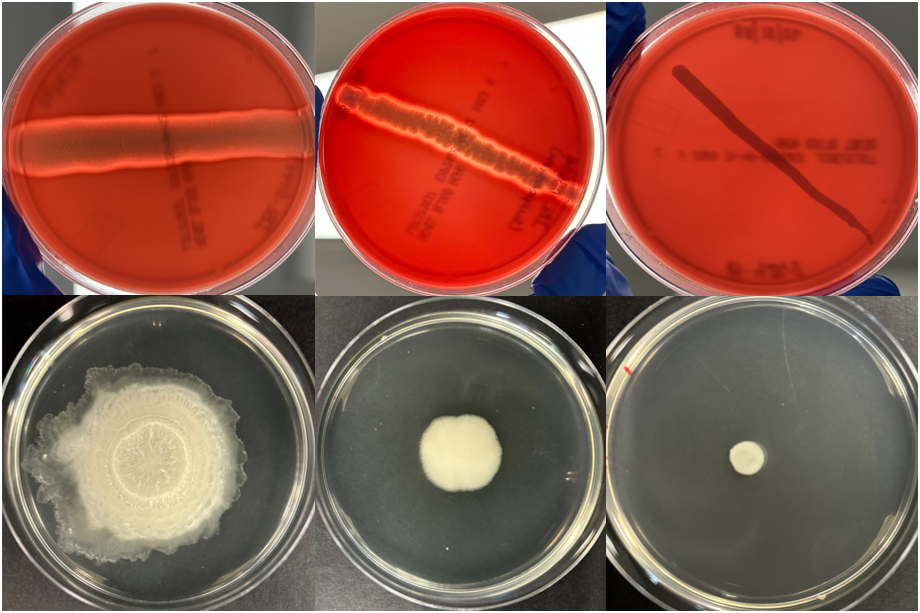


**Supplementary Fig S1.** **Hemolytic activity and motility of *Bacillus silvicola* IBL03679^T^.** Growth on blood agar plates after 24 h (top row) demonstrate beta-hemolytic activity of *Bacillus thuringiensis* HD-1 (left, positive control), *Bacillus silvicola* IBL03679^T^ (center), and no hemolytic activity of *Staphylococcus epidermidis* B-4268 (right, negative control). Colonies on 0.3% LB agar plates after 48 h (bottom row) demonstrate motility of *Bacillus thuringiensis* HD-1 (left, positive control), *Bacillus silvicola* IBL03679^T^ (center), and no motility of *Staphylococcus epidermidis* B-4268 (right, negative control). All plates grown at 30 ºC.


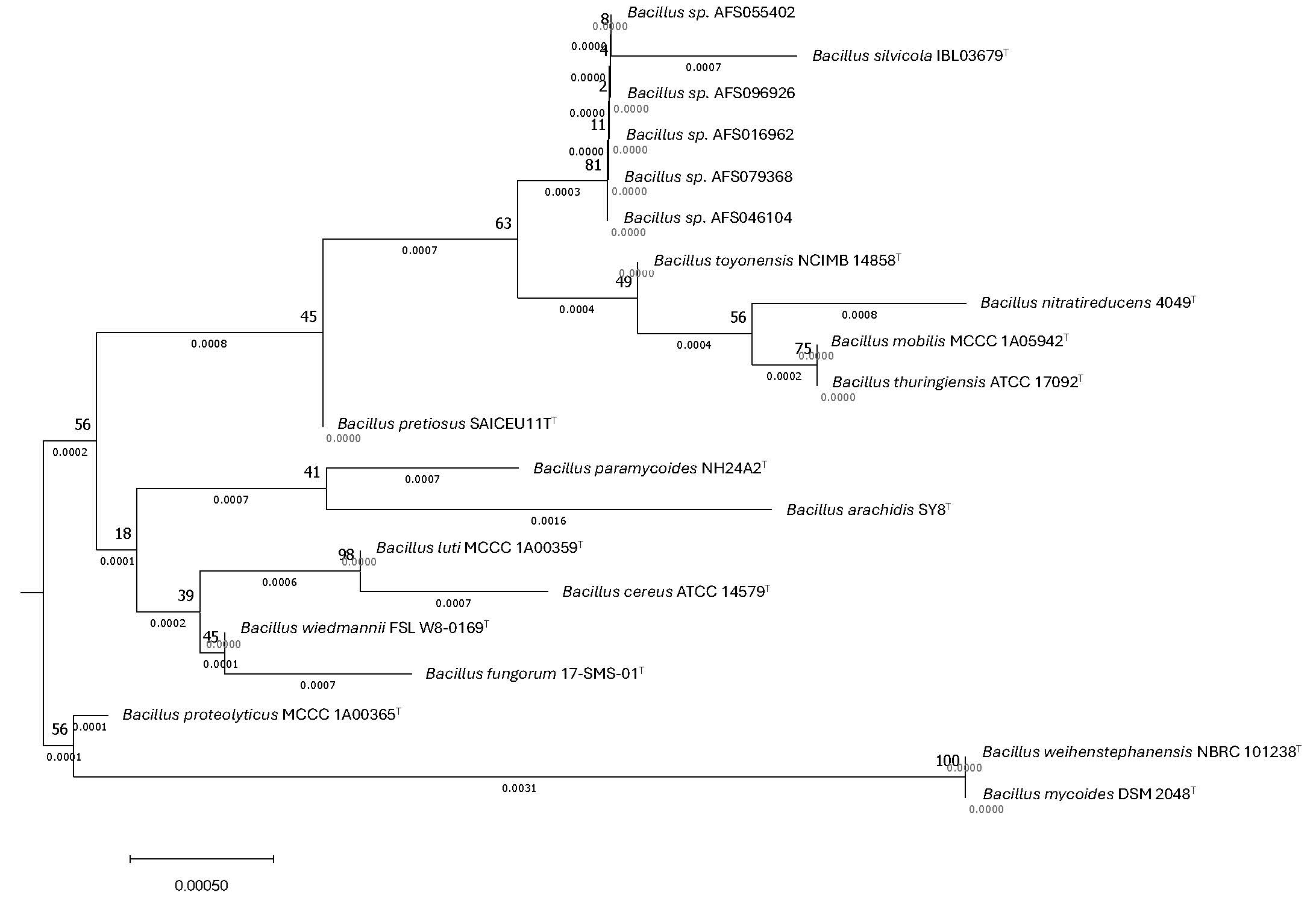


**Supplementary Fig S2. 16S-based phylogram of *B. silvicola* IBL03679^T^ and other related type-species.** Tree inferred using GBDP distances calculated from 16S rRNA gene sequences. Branch lengths are scaled in terms of GBDP distance formula *d_5_*. The numbers above branches are GBDP pseudo-bootstrap support values > 60% from 100 replications, with an average branch support of 46.5%. The tree was rooted at the midpoint


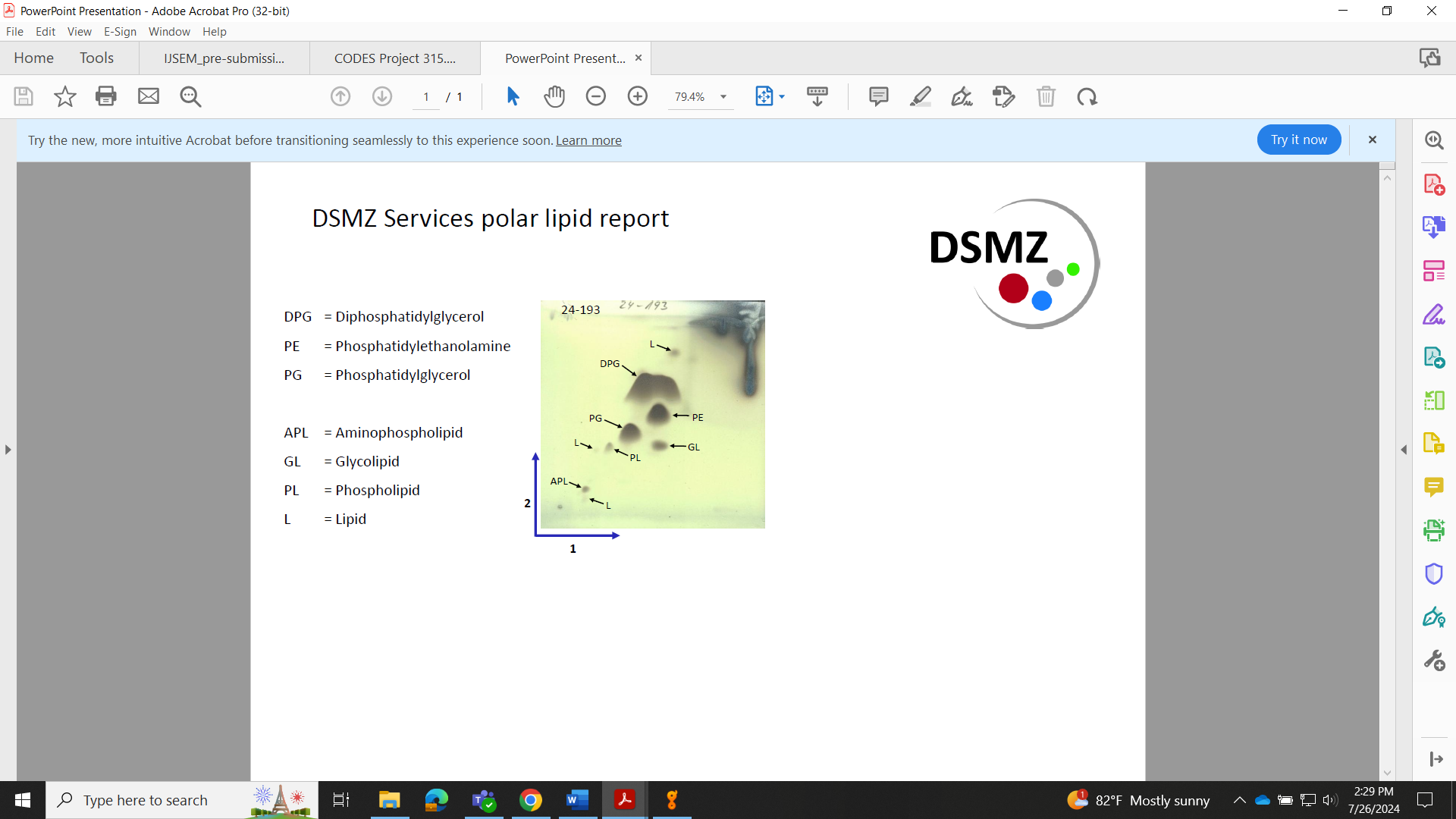


**Supplementary Fig S3.** **Polar lipid report for *B. silvicola* IBL03679^T^**
